# The evolution of human influenza A viruses from 1999 to 2006: A complete genome study

**DOI:** 10.1186/1743-422X-5-40

**Published:** 2008-03-07

**Authors:** Karoline Bragstad, Lars P Nielsen, Anders Fomsgaard

**Affiliations:** 1 Laboratory of Virus Research and Development, Statens Serum Institut, DK 2300 Copenhagen, Denmark; 2 WHO National Influenza Centre, Statens Serum Institut, DK-2300 Copenhagen, Denmark

## Abstract

**Background:**

Knowledge about the complete genome constellation of seasonal influenza A viruses from different countries is valuable for monitoring and understanding of the evolution and migration of strains. Few complete genome sequences of influenza A viruses from Europe are publicly available at the present time and there have been few longitudinal genome studies of human influenza A viruses. We have studied the evolution of circulating human H3N2, H1N1 and H1N2 influenza A viruses from 1999 to 2006, we analysed 234 Danish human influenza A viruses and characterised 24 complete genomes.

**Results:**

H3N2 was the prevalent strain in Denmark during the study period, but H1N1 dominated the 2000–2001 season. H1N2 viruses were first observed in Denmark in 2002–2003. After years of little genetic change in the H1N1 viruses the 2005–2006 season presented H1N1 of greater variability than before. This indicates that H1N1 viruses are evolving and that H1N1 soon is likely to be the prevalent strain again. Generally, the influenza A haemagglutinin (HA) of H3N2 viruses formed seasonal phylogenetic clusters. Different lineages co-circulating within the same season were also observed. The evolution has been stochastic, influenced by small "jumps" in genetic distance rather than constant drift, especially with the introduction of the Fujian-like viruses in 2002–2003. Also evolutionary stasis-periods were observed which might indicate well fit viruses. The evolution of H3N2 viruses have also been influenced by gene reassortments between lineages from different seasons. None of the influenza genes were influenced by strong positive selection pressure. The antigenic site B in H3N2 HA was the preferred site for genetic change during the study period probably because the site A has been masked by glycosylations. Substitutions at CTL-epitopes in the genes coding for the neuraminidase (NA), polymerase acidic protein (PA), matrix protein 1 (M1), non-structural protein 1 (NS1) and especially the nucleoprotein (NP) were observed. The N-linked glycosylation pattern varied during the study period and the H3N2 isolates from 2004 to 2006 were highly glycosylated with ten predicted sequons in HA, the highest amount of glycosylations observed in this study period.

**Conclusion:**

The present study is the first to our knowledge to characterise the evolution of complete genomes of influenza A H3N2, H1N1 and H1N2 isolates from Europe over a time period of seven years from 1999 to 2006. More precise knowledge about the circulating strains may have implications for predicting the following season strains and thereby better matching the vaccine composition.

## Background

Every year the influenza A virus causes human infection with varying severity depending on the host acquired immunity against the particular virus strain. Three to five million people experience severe illness and 0.25 to 0.5 million people die of influenza yearly worldwide (WHO EB111/10). The influenza virus evades host immunity by accumulation of point mutations (drift) in the major surface glycoproteins, haemagglutinin (HA) and neuraminidase (NA) or by reassortment of segments from different viruses co-infecting the same cell leading to a new stain with a HA (and NA) not seen in the population before (shift). In the worst case, shifts may cause pandemics. There have been three pandemics the last hundred years, the Spanish flu in 1918 (H1N1), the Asian flu in 1957 (H2N2) and the Hong Kong flu in 1968 (H3N2). It is believed that new pandemics emerge through shifts with strains from the avian reservoir, as was the case of the pandemics of 1957 and 1968, or by direct introduction of an avian strain into the human population as suggested for the 1918 pandemic [1]. At present only two of the 16 possible HA subtypes (H1 and H3), and two of the nine possible NA subtypes (N1 and N2) are circulating in man. H3N2 and H1N1 influenza A viruses have co-circulated in the human population since the re-emergence of H1N1 in 1977, increasing the possibility for genetic reassortments. The prevalence of the different subtype combinations may vary from season to season. The H3N2 has been the predominant influenza A strain during the last 20 years, with the exception of the 1988–1989 and 2000–2001 seasons where H1N1 infections dominated [2]. In the 2000–2001 season a new reassorted human strain, H1N2, emerged in Europe and became established in the autumn 2001 [3,4]. The new H1N2 subtype was covered by the 2002–2003 H1 and N2 trivalent vaccine components and because both H1 and N2 viruses had circulated the previous years some degree of herd immunity against the new strain was expected. The H1N2 viruses were not associated with severe influenza illness that season. In 2002, a new lineage A/Fujian/411/02(H3N2)-like emerged in Asia and caused significant outbreaks on every continent [5,6].

For the northern hemisphere the WHO issues the recommendation for strains to be included in the trivalent vaccine for the next season based on epidemiological data and antigenic and genetic analyses of circulating strains.

Until the recent release of over 1,800 complete influenza A genome sequences from the Influenza Genome Sequencing Project managed by US National Institute of Allergy and Infectious Diseases [7,8] very few complete genome sequences have been published to the GenBank. Also, there have been limited longitudinal studies of the complete genome of influenza A viruses. The present study characterise the complete genome evolution of H3N2, H1N1 and H1N2 influenza A virus from Denmark spanning seven seasons from 1999 to 2006.

## Results

### Prevalence of influenza A in Denmark from 1999 to 2006

**Figure 1:**
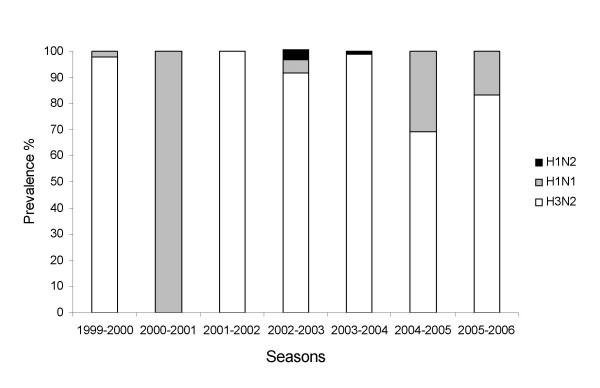
Relative prevalence of sentinel and routine influenza A viruses in Denmark 1999 to 2006. The actual numbers of influenza A positive samples for the respective seasons are as follows; 1999–2000 49, 2000–2001 28, 2001–2002 80, 2002–2003 61, 2003–2004 83, 2004–2005 91 and 2005–2006 54.

The relative prevalence of influenza virus varies from season to season. Influenza A H3N2 was the dominating strain in Denmark during the last seven years, with the exception of the 2000–2001 season where the H1N1 viruses dominated, as can be seen in Figure 1.

Only H3N2 viruses were isolated during the 2001–2002 season. In the 2002–2003 season the H3N2 and H1N1 reassorted influenza A virus strain, H1N2, emerged in Denmark, but has not been isolated in Denmark since 2003–2004. Higher prevalence of H1N1 viruses co-circulating with H3N2 viruses was observed the last two seasons, 2004/2005 and 2005/2006.

### Genetic evolution of influenza A

#### H3N2 viruses

**Figure 2:**
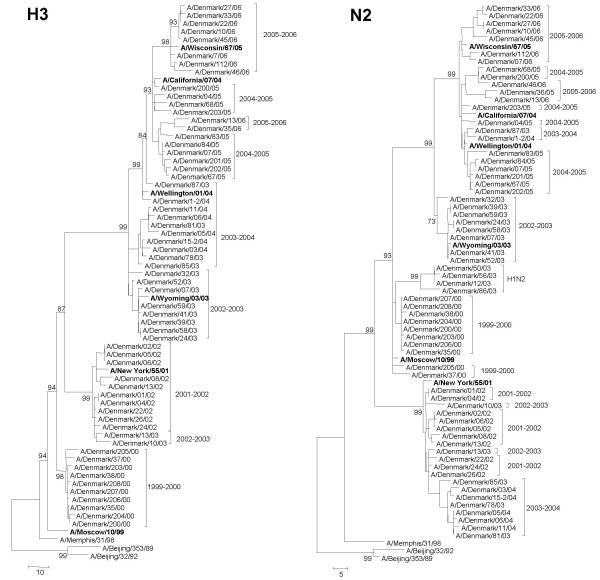
Evolutionary relationships of circulating H3N2 influenza A viruses sampled in Denmark from 1999 to 2006. The nucleotide coding region trees were generated with maximum parsimony, heuristic random branch swapping search (neighbor joining and maximum likelihood analysis revealed the same tree topology). Bootstrap values of 1000 resamplings in per cent (>70%) are indicated at key nodes. H3N2 HA and NA trees are rooted to A/Beijing/353/89 and A/Beijing/32/92. Reference sequences referred to in the text are shown in bold. The A/Fujian/411/02(H3N2) reference sequence is represented by A/Wyoming/03/03.

Based on phylogenetic analysis of the HA and NA nucleotide sequences from 1999 to 2006 (Figure 2), ten isolates representative for the phylogenetic clustering of sequences from each subtype in each season, as far as possible, were included in the final HA and NA tree (Figure 2) and representatives were chosen for complete genome sequencing. Generally the H3N2 HA and NA genes formed seasonal phylogenetic clusters (Figure 2). However, we observed that strains of different lineages and clusters co-circulated within the same season and that viruses had reassorted with viruses from previous seasons (Figure 2).

**Figure 3:**
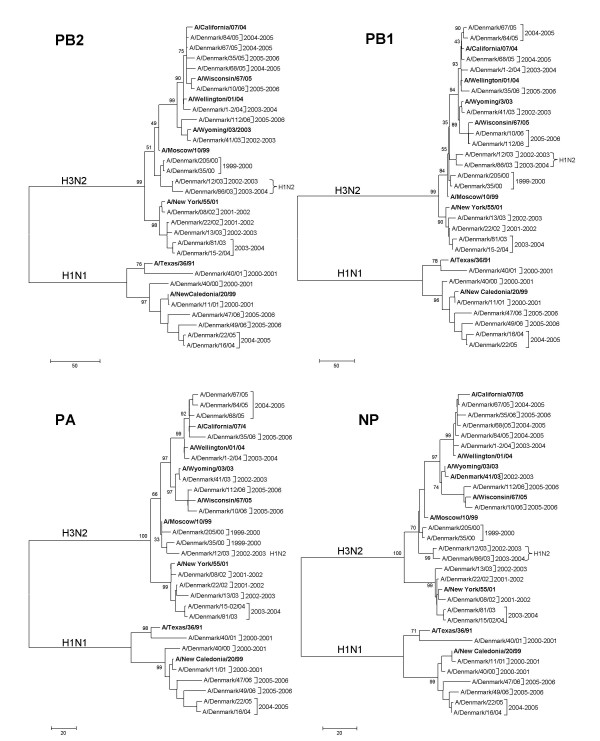
Evolutionary relationships of circulating H3N2 and H1N1 influenza A viruses sampled in Denmark from 1999 to 2006. The nucleotide coding region trees were generated with maximum parsimony, heuristic random branch swapping search (neighbor joining and maximum likelihood analysis revealed the same tree topology). Bootstrap values of 1000 resamplings in per cent (>70%) are indicated at key nodes. The trees for H3N2 and H1N1 PB2, PB1, PA and NP genes are mid-point rooted for means of clarity. Reference sequences referred to in the text are shown in bold. The A/Fujian/411/02(H3N2) reference sequence is represented by A/Wyoming/03/03.

The HA gene of the influenza H3N2 strains from the 1999–2000 season formed a phylogenetic subclade to A/Moscow/10/99(H3N2) and A/Sydney/5/97(H3N2) (represented by A/Memphis/31/98) (Figure 2), located between A/Moscow/10/99 and A/Panama/2007/99 (not shown). The antigenicity of these strains was A/Moscow/10/99(H3N2)-like in a haemagglutination inhibition assay, and will therefore be referred to as Moscow-like throughout this report. The NA and the internal genes were all A/Moscow/10/99(H3N2)-like, with the exception of the matrix (M) gene that clustered as a subclade to the A/New York/55/01-like strains (Figure 3).

The 2001–2002 season was represented as a monophyletic cluster of A/New York/55/01(H3N2)-like viruses in all genes (Figure 2). The next season, 2002–2003, was characterised by co-circulating lineages. These were of viruses most closely related to A/New York/55/01(H3N2) from the previous season, H1N2 viruses (described in more detail below) and a new H3 lineage, the A/Fujian/411/02(H3N2)-like viruses. The introduction of the A/Fujian/411/02(H3N2)-like viruses caused a "jump" in the evolution of the H3N2 viruses (Figure 2). The HAs in subsequent seasons have evolved from these viruses.

**Figure 4:**
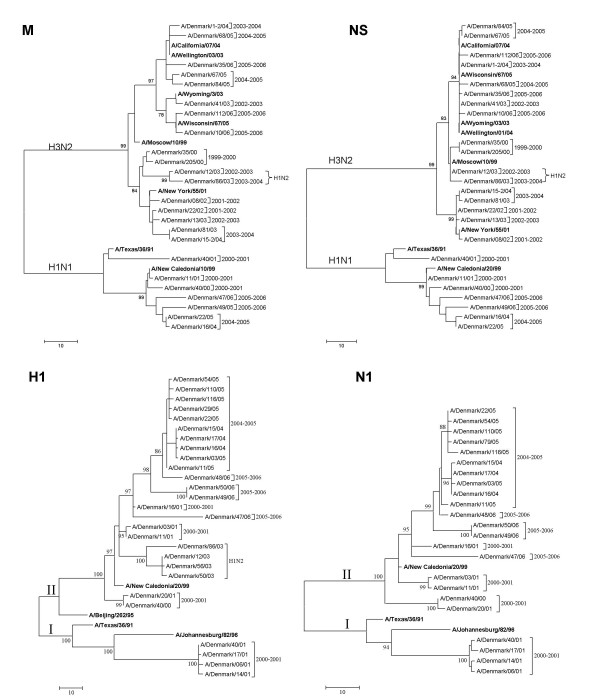
Evolutionary relationships of circulating H3N2 and H1N1 influenza A viruses sampled in Denmark from 1999 to 2006. The nucleotide coding region trees were generated with maximum parsimony, heuristic random branch swapping search (neighbor joining and maximum likelihood analysis revealed the same tree topology). Bootstrap values of 1000 resamplings in per cent (>70%) are indicated at key nodes. The trees for H3N2 and H1N1 M and NS and H1N1 HA and NA genes are mid-point rooted for means of clarity. Reference sequences referred to in the text are shown in bold. The A/Fujian/411/02(H3N2) reference sequence is represented by A/Wyoming/03/03.

In 2003–2004 the HAs form a subclade to the A/Fujian/411/02(H3N2)-like lineage from 2002–2003. These viruses were reassortants probably acquiring the rest of the genome from the 2001–2002 or 2002–2003 A/New York/55/01(H3N2)-like viruses (Figure 2, 3 and 4) and became the predominant lineage co-circulating with the A/Wellington/1/04(H3N2)-like viruses introduced from the southern hemisphere. One single H1N2 virus isolate was also observed this season. The A/Wellington/1/04(H3N2)-like lineage, the following season (2004/2005), had drifted into a more A/California/7/04(H3N2)-like lineage, causing a revision of the vaccine composition from A/Fujian/411/02(H3N2) to A/California/7/04(H3N2) [9]. In 2005–2006 the 2004–2005 A/California/7/04(H3N2)-like lineages continued to circulate together with the slightly different A/Wisconsin/67/05(H3N2)-like viruses (Figure 2). As a result the H3N2 vaccine component for the northern hemisphere 2006–2007 was changed to A/Wisconsin/67/05(H3N2) [9]. The A/Fujian/411/02(H3N2), A/Wellington/1/04(H3N2) and A/California/7/04(H3N2)-like viruses all share the same type of NS segments and there are few variations between the Wellington, California and Wisconsin-like strains, especially in the internal genes (Figure 3 and 4). However, the internal genes of the A/Wisconsin/67/05(H3N2)-like viruses, especially the polymerase acidic (PA), nucleoprotein (NP) and M are more closely related to the A/Fujian/411/02(H3N2)-like viruses from 2002–2003 than the A/California/7/04(H3N2) from the previous season (Figure 3 and 4).

#### H1N1 viruses

H1N1 viruses dominated the 2000–2001 season in Denmark (Figure 1). Thirteen isolates from this season were available for sequencing, and all were of the H1N1 subtype. These sequences represented two different co-circulating lineages (Figure 4). Lineage I is A/Bayern/7/95(H1N1)-like and lineage II include the H1N1 strains of today and the A/New Caledonia/20/99(H1N1) vaccine reference strain (Figure 4). The phylogenetic trees of NA and the internal genes showed the same topology (Figure 3 and 4). The lineage II strains are characterised by a deletion K130 in HA (K134 in H3 numbering) (Table 1). H1N1 virus was again isolated in 2004–2005 and showed a homogeneous distribution in the lineage II for all genes (Figure 3 and 4). Higher nucleotide variation was observed among the 2005–2006 H1N1 sequences in both HA and NA genes (Figure 4). This could indicate that the H1N1 viruses are in progression, away from the A/New Caledonia/20/99(H1N1)-like viruses.

**Table 1:** Amino acid changes in H3N2, H1N1 and H1N2 viruses between seasons *

**H3**							**N2**								**H1**						**N1**				
Amino acid	1999–00	2001–02	2002–03	2003–04	2004–05	2005–06	Amno acid	1999–00	2001–02	2002–03	2003–04	2004–05	2005–06	H1N2	Amino acid	H3 no.	2000–01	2004–05	2005–06	H1N2	Amino acid	N2 no.	2000–01	2004–05	2005–06

**5**	V	G	G	G	G	G	**18**	A		S(A)	A(S)	S	S		**43**	**53**	L(R)	L	L	L	**15**^Pa^	**15**	V/I	I	I
**25**	L		I(L)	I	I	I	**19**	T							**47**	**56**	I(T)	I	I	I	**23**	**23**	M/I	M	M
**33**	H	Q	Q	Q	Q	Q	**23**	L		F(L)	L(F)	F	F		**57**	**66**	V(I)	I	I	I	**45**^Pb^	**49**	H/Y	H	H
**45**^c^	S			I(S)			**24**	M						T	**69**^Cb^	**78**	L(S)	L	L	L	**48**	**52**	I(V)	I	I
**50**^c^	R		G(R)	G(E)	G	G	**30**	V		I(V)	V(I)	I	I		**71**	**80**	I(F)	I	I	I	**52**	**56**	R/K	R	R/K
**56**	H		H(Y)				**40**	Y			H(Y)				**80**	**88**	V(A)	V	V	V	**59**	**63**	S/R	S	S
**75**^E^	H		Q(H)	Q	Q	Q	**42**	C		F(C)	C(F)	F	F		**86**	**93**	E(K)	E	E	E	**64**	**68**	H/Q	H	H
**83**^E^	E		K(E)	K	K	K	**44**	S					S(P)		**89**	**96**	T			A(T)	**75**	**79**	V(I)	V	V
**92**^E^	T	K	K	K	K	K	**65**	I	I(T)						**94**	**100**	Y		H		**81**^Pc^	**81**	T/P	T	T
**106**	A	V	A(V)				**93**	N			N(D)	D	S(N/D)		**120**	**124**	D(E/G)	E	E	E	**83**	**83**	V/M	V	V
**112**	V				I(V)	V(I)	**143**	G		V(G)	G(V)	V	V		**130**	**134**	-(K)	-	-	-	**93**	**93**	S/P	S	S
**126**^a^	N			D(N)			**150**	H					H(G)		**133**	**136**	S(T)	S	S	S	**94**	**94**	I		I/V
**128**^b^	T		A(T)				**172**	K	R	K(R)	R(K)				**146**	**149**	R(K)	R	R	R	**95**	**95**	S/R	S	S
**131**^a^	A		T(A)	T	T	T	**194**	V					V(I)		**153**^Sb^	**156**	G(E)	G	G	G	**149**	**149**	V(I)	V	V
**144**^a^	I	D	N(D)	N(D)	N	N	**199**^b^	E			K	K	K	K	**163**^Sa^	**166**	K(M)	K	K	K	**155**	**155**	Y/F	Y	Y
**145**^a^	K				S/N	N	**216**	G		V(G)	G(V)	V	V		**166**^Ca1^	**169**	V(A)	A	A	A	**173**	**172**	K/R	K	K
**155**^b^	H		T(H)	T	T	T	**221**^b^	K	K(N)			K(D/E)	E		**168**	**171**	N(K)	N	N	N	**188**^Pd^	**187**	M/L	M	M
**156**^b^	Q		H(Q)	H	H	H	**258**	E						K	**170**^Ca1^	**173**	E(G)	E	E	E	**220**	**219**	K	K(E)	K
**159**^b^	Y			Y(F)	F	F	**265**	T	I		T(I)	I(T)			**175**	**177**	L			I	**222**	**221**	Q(R)	R	R
**164**	L	L(Q)					**267**	L	T	T	T	T	T	T	**179**	**181**	V		V(I)		**249**^Pf^	**248**	G/R	G	G
**173**^D^	K				E(K)	E(K)	**307**	V		I(V)	V(I)	I	I		**183**	**186**	P(S)	P	P	P	**254**	**253**	K/R	K	K
**186**^b^	S	G	G	G	G	G	**310**	Y					Y(H)		**185**	**188**	I(M)	I	I	I	**262**	**261**	K/R	K	K
**188**^b^	D				D(Y)		**329**^c^	N				N(T)	N(D)		**187**	**190**	D(N)	D	D	D	**270**	**269**	N/D	N	N
**189**^b^	S			S(N)	N	N	**332**^c^	S	F	S(F)	F(S)				**190**^Sb^	**193**	A			T	**274**	**273**	F/Y	F	F
**193**^b^	S					F(S)	**370**^c^	L					S(L)		**191**	**194**	L(I)	L	L	L	**332**^Pi^	**332**	E	K	K
**199**	S					S(P)	**372**	S				S(L)	S(L)		**194**	**197**	T(K)	T	T	T	**344**^Pj^	**347**	D(N)	D	D
**202**	V		I(V)	I	I	I	**385**^a^	K		N(K)	K(N)	N	N		**202**	**205**	V(L)	V	V	V	**352**^Pk^	**355**	K/R	K	K
**222**	W		R(W)	R	R	R	**392**^a^	I	I(T)						**215**	**218**	A			T	**364**	**367**	S(N)	S	S
**225**	G		D(G)	D	D	N(D)	**393**^a^	N		N(K)					**237**^Ca1^	**240**	G		G/R		**389**^Pm^	**397**	M/V	V	V
**226**^D^	V				I	I	**399**^a^	D	E	D(E)	E(D)				**239**	**242**	T(S)	T	T	T	**396**^Pm^	**399**	I/M	I	I
**227**^D^	S			S(P)	P	P	**401**^a^	G						D	**252**	**255**	W	R	R	W	**418**	**418**	I/M	I	I
**271**	N	D	D	D	D	D	**431**	K						N	**253**	**256**	Y	F	F	Y	**432**^Pn^	**432**	R(K)	R	R
**304**^c^	A	A/P	A(P)				**432**	Q	E	Q(E)	E	E	E		**267**	**269**	T(I)	T	T	T	**450**	**450**	N	D	D
**347**	V	M	V(M)				**437**	L	W	L(W)	W(L)				**271**	**273**	P(S)	P	P	P	**452**	**452**	D/E	D	D
**361**	T				I	I									**273**	**275**	D(G)	D	D	D					
**375**	N				D(N)	D(N)									**310**	**312**	A(T)	A	A	A					
**386**	E		G(E)	G	G	G									**315**	**317**	V	A	V	V					
**450**	R					R/K									**321**	**323**	I(V)	I	I	I					
**452**	R	K	K	K	K	K									**345**	**347**	V(I)	V	V	V					
**479**	G		E(G)												**382**	**384**	V(I)	V	V	V					
**529**	V	I	V(I)												**398**	**400**	N	S	N	N					
**530**	V		A(V)	A	A	A(V)									**451**	**453**	S(T)	S	S	S					
															**473**	**475**	N	D	D	N					
															**491**	**493**	E(K)	E	E	E					
															**506**	**508**	E			D					
															**510**	**511**	V(I)	V	V	V					

* Amino acids in brackets indicate less than half but more than two substitutions at the given amino acid position within a season. A single amino acid change in one position is not shown. Amino acids separated by '/' indicate equal substitutions of either amino acid at the given position. Letters in upper case above an amino acid indicate the antigenic site location of the residue. In N1 the upper case letter '^P^' stands for phylogenetically important region (PIR) and the following letters indicate the actual PIR.

#### H1N2 viruses

In 2002–2003 the reassorted H1N2 subtype combination was isolated for the first time in Denmark. The HA was derived from A/New Caledonia/20/99(H1N1)-like lineage II strains and the rest of the genome from A/Moscow/10/99(H3N2)-like viruses from the 1999–2000 season (Figure 2, 3 and 4). One single H1N2 sample was collected in the following 2003–2004 season and none have been sampled since in Denmark.

### Variations in the haemagglutinins

#### Variation among H3N2 viruses

The amino acid positions in H3N2 HA that have become fixed after 1999–2000 are G5, Q33, K92, G186, D271 and K452 (Table 1). After 2002, positions I25, Q75, K83, T131, T155, H156, I202, R222 and G386 have been stable (Table 1). Positions 50, 144, 145 and 225 had the highest variability represented by three different amino acids (Table 1).

The 2002–2003 season A/Fujian/411/02(H3N2)-like strains possessed eight substitutions at antigenic sites in HA compared to the strains of the previous A/New York/55/01(H3N2)-like season (Table 2) and the highest ratio of change was seen for antibody antigenic site B. This indicate that the preferred antigenic site in the change to A/Fujian/411/02(H3N2)-like strains was site B (*P*_epitope _= 0.190). The A/California/7/04(H3N2)-like strains from 2004–2005 showed changes at seven positions in the B-cell antigenic sites compared to the A/Fujian/411/02(H3N2)-like strains (Table 2) and again the preferred antigenic site for change was site B (*P*_epitope _= 0.143). Several changes were also observed at antigenic site D (*P*_epitope _= 0.073) (Table 2).

**Table 2:** Amino acid variations at antibody antigenic sites in HA (A-E) and NA (A-C) of H3N2 viruses 1999 to 2006

Haemagglutinin					Neuraminidase			
Antigenic site	Moscow-New York-like	New York-Fujian-like	Fujian-California-like	California-Wisconsin-like	Moscow-New York-like	New York-Fujian-like	Fujian-California-like	California-Wisconsin-like
**A**	I144D	A131T D144N	K145N		D399E	K385N E399D		
**B**	S186G	T128A H155T Q156H	A128T Y159F S189N	S193F			E119K K221E	
**C**		R50G			S332F	F332S		L370S
**D**			K173E V226I S227P	E173K				
**E**	T92K	H75Q E83K						

The H3 strain component of the 2006–2007 influenza vaccine for the northern hemisphere was A/Wisconsin/67/05(H3N2). We measured the rate of change at antigenic sites between the A/California/7/04(H3N2)-like viruses from 2004–2005 and the 2005–2006 A/Wisconsin/67/2005(H3N2)-like viruses. Only two substitutions at HA antigenic sites defined the A/Wisconcin/67/2005(H3N2)-like viruses (Table 2). Amino acids at positions 225 to 227 in H3 have greatly changed the last seasons (Table 1). Position 226 and 227 are directly involved in the antigenic site D.

Since the introduction of Fujian like strains in 2002–2003 there have been substitutions at sites that may influence the capacity for egg growth; 131, 155, 156, 186, 222, 225 and 226 (possibly also positions 144, 145, 159 and 193) [10] (Table 1). Amino acids 193, 222, 225, 226 and 227 are involved in receptor binding sites in the HA, therefore the changes observed at these sites in our dataset may influence receptor binding. Amino acids defining the T-cell epitopes (after the list of Suzuki [11]) in HA have remained unchanged since 1999.

#### Variation among H1N1 viruses

The phylogenetic H1N1 lineage II is characterised by an amino acid deletion K130 (position 134 in H3 numbering) (Table 1) and certain amino acid differences in the antibody antigenic sites; substitution M166K in the antigenic site Sa, E156G in site Sb, V169A and G173E in site Ca1 and substitution S78L at site Cb (H3 numbering) (Table 1). The calculated *P*_epitope _values indicate that antigenic site Ca1 has been the site with the largest proportion of substitutions (*P*_epitope _= 0.500) in the change from H1 lineage I to lineage II (Table 1). Some isolates from 2005–2006 possessed an additional change V181I. One change, G240R, found in two of four isolates from 2005–2006, is positioned in the Ca1 antigenic site (Table 1).

#### The H1N2 viruses

The HA gene of the Danish H1N2 viruses belong to the H1N1 A/New Caledonia/20/99(H1N1)-like lineage II with the K134 deletion. The HA from the H1N2 reassorted strains possessed one additional substitution in the antibody antigenic sites of HA, A193T (H3 numbering) site Sb, compared to other HAs from lineage II H1N1 viruses. Other amino acids that characterised the HA H1N2 viruses were: A96, I177, T218 and D508 (H3 numbering) (Table 1).

### Variations in the neuraminidases

The amino acid change L267T in the N2 neuraminidase has become fixed after 1999. NAs from 2004 to 2006 all possess K199 and E432. Comparing consensus sequences of the different phylogenetic clusters it is clear that after 1999 there have been changes at the antibody antigenic sites of NA (Table 2). In addition, two out of ten H3N2 isolates from 2004–2005 and three out of ten H3N2 isolates from the 2005–2006 seasons differed also at antibody antigenic site C N329T and N329D, respectively, compared to the seasons before. The NAs of the H1N2 viruses were most closely related to the 1999–2000 seasons A/Moscow/10/99(H3N2)-like viruses but varied at six amino acid residues: M24T, E199K, E258K, L267T, G401D and K431N (Tabel 1). Position K199 found in antigenic site B, D401 in antigenic site A and N431 may influence antigen binding. The observed changes at site 93 in N2 from 2003 to 2006 (Table 1) are located in the HLA-A*0201 NA_90–99 _(PQCNITGFAP) CTL epitope [12].

For the N1 viruses there have been several changes at phylogenetically important regions (PIRs) [13] (Table 1). Changes were observed at regions equivalent to the N2 antigenic sites, namely: PIR-I E332K, PIR-J N344D, PIR-K R352K, PIR-M M389V and M396I, and PIR-N K432R.

No genetic indication of neuraminidase drug resistance at positions 119, 152, 274, 292 or 294 was found in the NA dataset from 1999 to 2006.

### Variations in the internal genes

The substitution PB2 (polymerase basic 2 protein) S569A in the H3N2 sequences has become fixed after the 1999–2000 season (not shown). All H3N2 isolates from 2004 to 2006 have changed at position V709I in the PB1 protein.

The lineage I H1N1 PA protein possessed the amino acid C226 (as did the H3 isolates) instead of I226 found in the H1 lineage II isolates. This position is part of a HLA-A*0201 PA_225–233 _(CLENFRAYV) T-cell epitope [12]. Also the substitution V602I is located in the HLA-B*8 PA_601–609 _(SVKEKDMTK) CTL epitope [14] for all H1N1 viruses and the H3N2 2005–2006 season viruses.

The T146A substitution in the H3N2 NP protein has become fixed after 1999–2000 season. The substitution NP Y52H found in the A/California/7/04(H3N2)-like isolates from 2004 to 2006 is located in a CTL epitope HLA-A*01 NP_44–52 _(CTELKLSDY) [15]. The H1N1 isolates possessed a S50N replacement in this epitope. The H3 A/New York/55/01(H3N2)-like isolates from 2001–2002 and 2002–2003 together with the Fujian/New York reassortants from 2003–2004 possessed K98 in the HLA-A*6801 NP_91–99 _(KTGGPIYRR) [16]. Also H1N1 strains from the lineage II possessed this change. The isolates from 1999–2000 and after 2002 possessed the "original" CTL epitope HLA-B*1508 NP_103–111 _(KWMRELVLY) [17] while the 2001–2002 viruses possessed a K103R replacement. This replacement was also seen in some 2003–2004 isolates. All H1N1 isolates have the M105V replacement. The HLA-B*4002 NP_251–259 _(AEIEDLIFL) epitope has been conserved in the H3N2 and H1N2 isolates. The H1N1 isolates have a I257T substitution.

After 1999 CTL epitope HLA-B*1402 NP_146–154 _(TTYQRTRAL) [18] has changed with the substitution T146A in the H3 isolates, all H1 viruses still possess T146. The New York/55/01(H3N2)-like viruses possessed the substitution V197I in the CTL epitope HLA-A*1101 NP_188–198 _(TMVMELIRMVK) [12] as did the H1N1 viruses. The H1N1 isolates also had a M191V change in this epitope region. The A/Wisconsin/67/05(H3N2)-like viruses from the 2005–2006 season changed in the CTL epitope HLA-DQA1*0501/HLA-DQB1*0201 NP_365–379 _(IASNENMDNMGSSTL) [19] with the substitution S377G. The H1N1 viruses had three amino acid differences in this epitope; N373A, M374I and G375V. All virus subtypes in this dataset had the R384G substitution in the CTL epitope HLA-B*27 NP_383–391 _(SPYWAIRTR) [14]. The A/Fujian/411/02(H3N2), A/California/7/04(H3N2) and A/Wisconsin/67/05(H3N2)-like viruses possess the substitution V425I in the CTL-cell epitope HLA-B*0702/HLA-B*3501 NP_418–426 _(LPFEKSTVM) [20] as did the H1N1 viruses. Two additional differences were observed in this region of the H1N1 viruses, E421D and S423T.

The H1N2 viruses differed in the M2 protein from the A/Moscow/10/99(H3N2)-like viruses with the amino acid substitutions; G16E, C17Y and N20S. The substitution V15I in the M1 protein located in the HLA-A*1101 M1_13–21 _epitope [12] was found in two of the H1 isolates from 2000–2001, one in lineage I and one in lineage II. The H3N2 and H1N2 viruses in this dataset before 2005–06 had the substitution R174K in CTL epitope HLA-B*39 M1_173–181 _(IRHENRMVL) [14]. The substitution S31N in the M2 protein of the A/Wisconsin/67/05(H3N2)-like 2005–2006 viruses indicates resistance to the influenza matrix ion channel inhibitory drug amantadine [21,22].

H3N2 NS1 (non-structural protein) amino acids that have become fixed after 1999 are K26E and E221K. The NS1 CTL epitope HLA-DR*03 NS1_34–42_(DRLRRDQKS) identified in H1N1 and H5N1 viruses [23] has the substitution K41R in the H3N2 viruses from this dataset. The HLA-A*0201 NS1_122–130 _(AIMDKNIIL) epitope identified in H1N1 viruses has the D125E and I129M amino acid differences in the H3N2 isolates. There has been a substitution, F166L, in the HLA-B*44 NS1 158–166 CTL epitope [24] for 2000–2001 H1N1 isolates in both lineage I and lineage II.

### Glycosylation patterns

**Figure 5:**
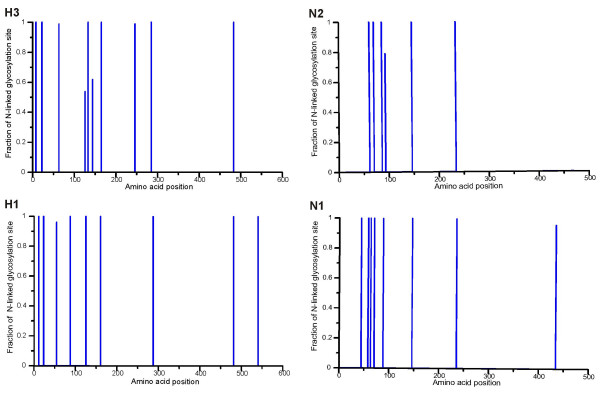
Fraction of predicted N-linked glycosylation sequons in HA and NA of H3N2 and H1N1 viruses sampled in Denmark seasons 1999 to 2006. Sites with predicted potential threshold values above 0.5 are shown. Sites not shown for H3 (n = 204): 122, N2 (n = 166): 200, 329, 402, H1 (n = 27): 10, position 539 is positively predicted; however this site is located at the cytosolic region of HA and is therefore not glycosylated, N1 (= 30): 455.

Eight potential N-glycosylation sites in H3 HA1 have been constant since 1999, namely: 8, 22, 63, 133, 165, 246, 285 in H1 and 483 in HA2 (Figure 5). These glycosylation sites have been conserved in our dataset from 1999 to 2006. The A/Moscow/10/99(H3N2)-like viruses from the 1999–2000 season possessed two additionally predicted sites 38 and 126. The A/New York/55/01(H3N2)-like viruses from the 2001–2002 season had lost the position 38 sequon but possessed the potential glycosylation site at position 126. The position 38 sequon was observed after 1999–2000, but the predicted score has been below the set threshold value of 0.5 and therefore not included in the count further (Figure 5). In 2002–03 two out of four A/New York/55/01(H3N2)-like viruses possessed ten potential glycosylation sites. Compared to the A/New York/55/01(H3N2)-like viruses from the season before, they gained a glycosylation at position 144. The A/Fujian/411/02(H3N2)-like viruses from the 2002–2003 season possessed nine potential glycosylations, they kept the newly introduced sequon at position 144 but did not possess the 126 sequon (Figure 5). The 2003–2004 A/Fujian/411/02(H3N2)-like reassorted viruses had the same glycosylation pattern as the previous season Fujian-like viruses. However, Fujian-like viruses that neither possessed the 126 nor the 144 potential glycosylation sequons were also observed, resulting in a total of eight potential sites only. The A/Wellington/1/04(H3N2)-like viruses from 2003–2004 season possessed ten potential glycosylation sites. In addition to the eight conserved they had glycosylation sites at position 126 and 144. The A/California/7/04(H3N2) and A/Wisconsin/67/05(H3N2)-like viruses from 2004 to 2006 have the same ten glycosylation sites as the A/Wellington/1/04(H3N2)-like viruses. Both position 126 and 144 are located at HA antigenic site A.

Six potential N-linked glycosylation sites were predicted for N2 strains from 1999 to 2003, namely: 61, 70, 86, 93, 146 and 234 (Figure 5). In the 2003–2004 season a minority of the isolates and all of the isolates the following season had lost the potential site 93. The 93 predicted sequon was seen again in three (A/Wisconsin/67/05(H3N2)-like) out of ten isolates from the 2005–2006 season. The same glycosylation pattern was observed for the NA of both H3N2 and H1N2 sequences.

The H1 HA strains from 1999 to 2006 have seven predicted potential N-linked glycosylation sites in HA1 (11, 23, 54, 87, 125, 159, 286) and one in HA2 (480) (H1 lineage II numbering) (Figure 5). The exception is one single isolate (A/Denmark/03/05) from 2004–2005 which lacked the sequon at position 54. The isolates in lineage I possessed one additional sequon at position 268; however the calculated potential was below the threshold value of 0.5 and therefore not counted (Figure 5). Three out of four HA H1N2 sequences lacked the position 87 sequon.

The eight potential glycosylation sites in the N1 NA protein have been conserved since 1999 (positions 44, 58, 63, 70, 88, 146, 235 and 434).

### Sequence data

**Figure 6:**
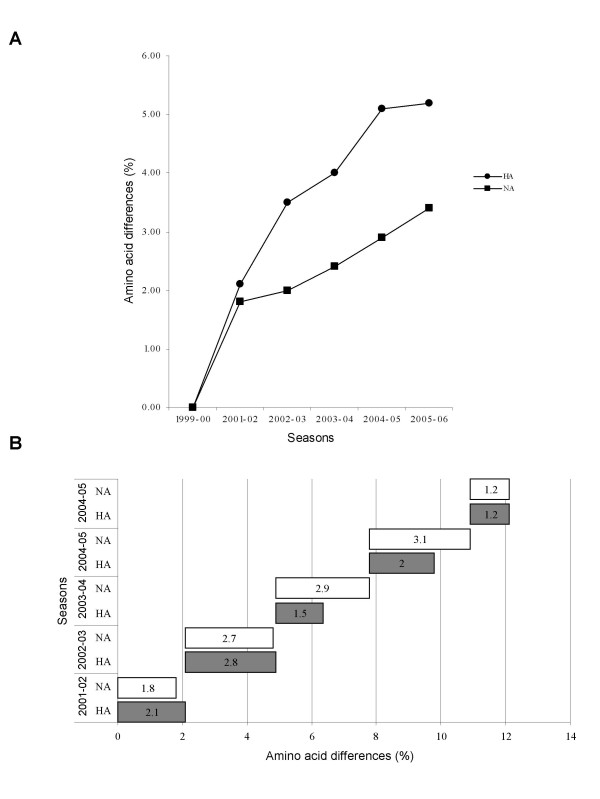
(A) Seasonal amino acid distances of H3N2 HA and NA proteins since 1999 and (B) amino acid distances of H3N2 HA and NA from one season to the next. The same trends were observed for nucleotide distances. In 2000–2001 H1N1 viruses only were observed and therefore not included. Distance means were computed as the arithmetic mean of all pair wise distances between two seasons in the inter-season comparisons by the MEGA v.3.1 software [68]

The proposed "jump" in the evolution of H3 with the introduction of the A/Fujian/411/02(H3N2)-like viruses in 2002–2003 can also be seen in Figure 6. Rather than constant drift the genes have evolved in a more stochastic like process (Figure 6). The evolution of H3N2 viruses was influenced by a reassortment event in 2003–2004 where the drifted A/Fujian/411/02(H3N2)-like viruses acquired a A/New York/55/01(H3N2)-like backbone from viruses that circulated in 2001–2002 and to some extent in 2002–2003 (Figure 2, 3 and 4). The 2003–2004 viruses had few variations in the HA compared to the previous season and the 2005–2006 viruses had few variations compared to viruses circulating the season before, both in HA and NA genes (Figure 6B). These stasis-periods might indicate a well fit virus in less need for change.

Based on the ratio of non-synonymous versus synonymous substitutions none of the influenza A genes were directly influenced by positive selection (dN/dS<1) (Table 3). However, as expected the HA1 region of both H3 and H1 viruses were more influenced by evolutionary pressure (Table 3). We applied FEL and SLAC maximum-likelihood methods to estimate individual positively selected sites in H3N2 HA and NA and added REL for smaller datasets in all genes (se methods section). The FEL method found one site in the H3 protein (n = 204), position 199 (*p *= 0.046) to be positively selected, while the more conservative SLAC analysis found none. No positive selected sites were predicted for the N2 genes (n = 166) and none in the internal genes (n = 15) estimated by FEL and SLAC. The REL analysis retrieved four sites in the M1 gene to be selected namely positions 208, 211, 218 and 219. No sites in HA (n = 27) and NA (n = 30) of the H1N1 viruses were directly positively selected with any of the three methods of analysis.

**Table 3:** Non-synonymous/synonymous substitution ratio for H3N2 and H1N1 isolates sampled in Denmark from 1999 to 2006*

**Gene**	**dN/dS**	
	H3N2	H1N1^a^
HA	0.23	0.18
HA1	0.53	0.21
HA2	0.11	0.08
NA	0.25	0.17
PB2	0.06	Na
PB1^b^	0.07	Na
PA	0.08	Na
NP	0.08	Na
M1	0.07	Na
M2	0.38	Na
NS1	0.23	Na
NS2	0.19	Na

* dN/dS is the ratio of nonsynonymous vs. synonymous substitutions calculated by SLAC likelihood analysis.

^a ^The internal genes of H1N1 viruses were not included due to the few sequences available (Na). H3 n = 204, N2 n = 166, internal genes n = 15, H1 n = 27, N1 n = 30.

^b ^dN/dS for PB1-F2 was not included because the method used was biased by the PB1-F2 +1 reading frame from PB1. Synonymous substitutions in PB1 would therefore be assigned as non-synonymous in PB1-F2 not reflecting positive selection on the PB1-F2 itself.

## Discussion

### Prevalence of influenza A from 1999–2006 in Denmark

The H3N2 strains have had the highest prevalence since 1999 in Denmark. The H3N2 strains have undergone more changes in the antigenic sites each season in HA and NA than have H1N1 strains, thereby evading the host immune system more efficiently than H1N1. The exception is the 2000–2001 season which was dominated by a newly introduced H1N1 strain, the lineage II A/New Caledonia/20/99(H1N1)-like viruses, antigenically different from the previous lineage I-like viruses (A/Bayern/7/95(H1N1)-like).

In 2002–2003 the H1N2 reassorted strain was observed in Denmark for the first time. This strain probably emerged in South Asia in 2001 and spread to many countries [4,25,26] The H1N1 component in the vaccine for that season matched the H1 and the H3N2 component matched the N2 subtypes of the reassortant strain. Thereby, the new strain was expected fully covered by the vaccine for that season [26] and it was anticipated that there would be some extent of herd immunity in the population against this new reassortant.

### Genetic evolution of influenza A

The phylogenetic trees of H3N2 HA and NA showed seasonal clusters but also co-circulating lineages within seasons. The introduction of the A/Fujian/411/02(H3N2) strains in 2002–2003 caused a "jump" in the evolution of both HA and NA genes. Many of the substitutions in HA introduced with the A/Fujian/411/02(H3N2)-like viruses have become fixed, probably reflecting a very fit virus. The genetic variation before the 2003–2004 season may have been more influenced by introduction of new viruses through viral migration than adaptive evolution of the genes. A constant rate of drift was not observed for HA but instead periods of change followed by stasis periods. The low dN/dS ratios (0.232 for HA and 0.247 for NA) also indicated that the influenza genes were not directly influenced by positive selection. Reassortments between co-circulating strains and viruses from previous seasons, and introduction of viruses from other parts of the world might play a larger role than natural selection for some seasons, as also observed by others [27,28].

The A/Fujian/411/02(H3N2)-like viruses, genetically and antigenically different from the previous seasons, were first seen in Denmark in 2002–2003. The 2002–2003 season was clearly a turning point in regard to circulating influenza A H3N2 viruses in Denmark and caused a mismatch in the herd immunity of the population and in the protection of the seasonal influenza vaccine containing the A/Moscow/10/99(H3N2) strain [29]. The fitness of the Fujian-like viruses in Denmark was increased by a reassortment event in 2003–2004 when drifted HA A/Fujian/411/02(H3N2)-like viruses acquired the rest of the genome from A/New York/55/01(H3N2)-like viruses seasons before. The reassortment has also been observed for isolates from New York State [28]. This reassorted strain caused the only epidemic in the study period in Denmark. The increased fitness could have been a result of a more effective New York-like NA, compared to the weak Fujian-like NA circulating the season before shown by others [30]. The HA genes from viruses circulating after 2002–2003 are derived from the A/Fujian/411/02(H3N2)-like viruses. The viruses circulating in the 2005–2006 season had few variations in HA and NA compared to the viruses circulating the season before. However; the internal genes of the A/Wisconcin/67/05(H3N2)-like viruses, especially PA, NP and M, were more realated to the A/Fujian/411/02(H3N2)-like viruses rather than the previous seasons A/California/07/04(H3N2)-like viruses.

Two H1N1 lineages, lineage I (A/Bayern/7/95(H1N1)-like) and II (A/New Caledonia/20/99(H1N1)-like), co-circulated in the 2000–2001 season in Denmark. A variant of A/Beijing/262/95(H1N1)-like viruses with the K130 deletion was isolated in New Caledonia in the southern Pacific in 1999, A/New Caledonia/20/99(H1N1)-like viruses (lineage II). These viruses replaced the A/Bayern/7/95(H1N1) lineage (lineage I) [31] as was also the case in Denmark. Subsequently the northern hemisphere vaccine composition was changed from A/Beijing/262/95(H1N1) to A/New Caledonia/20/99(H1N1) for the 2000–2001 season [32]. Since the introduction of the A/New Caledonia/20/99(H1N1)-like viruses to Denmark in 2000–2001 there have been few substitutions in the glycoproteins. This is also seen in the rest of the world and the H1N1 component in the trivalent vaccine recommended for the northern hemisphere has been A/New Caldonia/20/99(H1N1) since 2000–2001. The greater variability of the 2005–2006 H1N1 viruses observed in the phylogenetic trees possibly indicates that the virus is changing. If the new viruses are able to evade the host immune system more efficiently, H1N1 may become the prevalent strain again in near future.

The reassorted H1N2 strain was first observed in Denmark in 2002–2003, while circulating in many parts of the world in 2001–2002 [2,4,25,26]. The reassorted H1N2 viruses possessed only the HA from A/New Caldonia/20/99(H1N1)-like viruses and the rest of the genome from A/Moscow/10/99(H3N2)-like viruses as also reported by others using partial sequences [25]. The H1N2 viruses have been introduced to Denmark from elsewhere and are not a local reassortant.

### Amino acid changes in the haemagglutinins

Key positions in H3 HA for antibody antigenic change are 144 and 145 located in antigenic site A. Changes in the 140–146 region, antigenic site A, is characteristic for antigenically distinct viruses of epidemic significance [33]. Post-infection sera contain antibodies that recognise mainly residue 144 but also residue 198 and 157 on site B [34,35]. Position 144 changed from I144 to D144 between 1999–2000 and 2001–2002 and further to N144 with the introduction of the A/Fujian/411/02(H3N2)-like viruses in 2002–2003. This important residue (N144) has hereafter remained unchanged. Several amino acid substitutions introduced with the A/Fujian/411/02(H3N2)-like viruses were retained the subsequent seasons (Table 1) suggesting they may be important for viral escape from the host immune system and the overall fit of the virus.

The A/Fujian/411/02-like(H3N2) viruses did not antigenically match the A/Moscow/10/99(H3N2) strain included in the 2002–2003 vaccine [29,36]. The HA substitution D144N was however not responsible for the antigenic drift of the Fujian-like viruses. It has been shown that only two amino acid changes, H155T and Q156H, specified the antigenic difference from Moscow-like to Fujian-like [37], both are located at antigenic site B. The T155 and H156 amino acids have been maintained in all Danish isolates after the introduction of the Fujian-like viruses. With the A/California/7/04(H3N2)-like viruses in 2004–2005, site 145 has changed from K to S in some isolates and to N in others. S145 has been found at this position before 1999 and N145K was the only cluster-difference substitution between isolates from the seasons 1987/1989 and 1992/1995 [38]. It was therefore suggested that substitutions at this site alone could have large antigenic effect. Antigenic site A, located in a loop, makes few contacts with the rest of the structure, therefore 144 and 145 may change drastically without influencing on the overall shape of the HA molecule.

Antigenic site A is supposed to be ideal for antibody binding and for amino acid replacements [33]. The preferred antigenic site in the change from A/Moscow/10/99(H3N2) to A/Fujian/411/02(H3N2)-like viruses was site B. This observation is in accordance with previously published data [39]. One region in HA, position 225 to 227, that influence antigenic site D, has changed drastically during the study period from GVS → DVS → DIP → NIP. The influence of antigenic site D was first apparent in the antigenic change from Fujian-like viruses to California-like viruses; however, the antigenic site B was still the preferred site for antigenic change. It has been proposed that a minimum of four substitutions in two or more antibody binding sites are required for an epidemically important strain [40]. The 2002–2003 A/Fujian/411/02(H3N2)-like viruses in Denmark had changed at eight positions in four HA antigenic sites and three changes in two NA antigenic sites compared to the 2000–2001 New York-like viruses. The change from Fujian-like to California-like viruses involved seven changes at three HA antigenic sites and two changes at one NA antigenic site. The vaccine composition was subsequently changed for the 2005–2006 season [9]. The 2006–2007 vaccine composition was again changed to include A/Wisconsin/67/05(H3N2). The observed difference from circulating Danish California-like to Wisconsin like viruses involved only two substitutions at two antigenic sites in HA and one in NA.

### Neuraminidase

Gulati *et al*., [41] have suggested that new antigenic variants may arise from changes that influence on N2 antigenic site B. The A/Fujian/411/02(H3N2)-like reassorted viruses from 2003–2004 introduced a E199K change in NA which since has become fixed in Danish isolates. The A/Wisconsin/67/05(H3N2)-like viruses from 2005–2006 introduced the K221E substitution, both found in the antigenic site B. Neuraminidase substitutions inducing resistance to neuraminidase inhibitory drugs like amantadine might include one or more of the amino acid residues 119, 152, 274, 292 and 294 (N2 numbering). The most frequent NA substitutions observed *in vitro *are the active site residues E119G/D/A and R292K [22,42-45]. The substitution H274Y is the only neuraminidase resistance mutation identified in H1N1 viruses to date [46,47]. Danish isolates did not possess any resistance mutations in the NAs. Neuraminidase inhibitory drugs are rarely used for influenza prophylaxis or treatment in Denmark.

### Internal proteins

T-cell epitopes are more conserved than antibody epitopes. Fifteen per cent of the T-cell epitopes are conserved while only 2.7% of the antibody epitopes [48]. The reason for this higher degree of conservation is that 80% of the antibody epitopes are located in the variable glycoproteins HA and NA, while only 40% of the T-cell epitopes are found in these proteins [48]. Recent research has shown some degree of escape from CTL-mediated immunity in addition to escape from neutralizing antibodies [28]. In our dataset we found several substitutions in regions involved in protective T-cell response [48] in NA, PA, M1, NS1 and most in the NP protein. This is not unexpected because most T-cell epitopes are defined for the NP protein and this protein is the main target for the cytotoxic host immune response [49,50]. The extensive variations in the T-cell epitopes during 1999 to 2006 suggest that these regions and the antibody epitopes are working together for efficient escape from the host defence responses.

The M2 proteins from the Danish Wisconsin-like viruses in 2005–2006 possessed the substitution S31N, associated with resistance to matrix inhibitory drugs, like amantadine [22,44,45,51]. These types of drugs are not used for prophylaxis or treatment in Denmark. The S31N substitution is therefore not a local introduced resistance mutation. We cannot exclude that this substitution has arisen by chance, but it is more likely that the mutation has emerged as a resistance mutation in other countries like the USA [52] and Australia [53] where the prevalence of amantadine resistance is high. The resistance may also be related to the increased use of this drug in Asia during the SARS epidemic [21].

### Variations in receptor specificity

The A/Fujian/411/02(H3N2)-like clinical Danish viruses had several substitutions in HA at sites that might influence the virus' capability for egg growth [10,37]. These include; A131T, I144N, H155T, Q156H, W222R and G225D. The A/California/20/99(H3N2)-like viruses had further changes at positions K145S/N, Y159F, S193F and V226I and A/Wisconsin/67/05(H3N2) possessed in addition S193F and D225N. All isolates after the 1999–2000 A/Moscow/10/99(H3N2)-like viruses possess S186G. In recent years H3N2 viruses have had poor replication efficiency in eggs [54,55]. It has been shown that positions 186, 226 and 196 are critical determinants for egg growth. The changes G186V and V226I increased egg viral replication of A/Fujian/411/02(H3N2) viruses so did the changes G186V and A196T for A/California/20/99(H3N2) viruses [56,57]. On the contrary, others have stated that the V226I change in combination with T155 and H156 do not result in viral recovery in eggs [37,54]. This might explain the delay in the 2006–2007 vaccine production for the northern hemisphere due to egg propagation difficulties with the A/Wisconsin/67/05(H3N2) strain. The influence on replication efficiency by the other substitutions observed at receptor binding sites should be investigated further.

We did not observe amino acids in the N2 NA protein that would decrease virus replication in eggs. The amino acids known to give good replication in eggs (Q119, K136 and Y347) [56] were all present in this dataset.

### N-linked glycosylation pattern

Oligosaccharides at the surface proteins HA and NA might have greater impact on viral escape from the immune system than single amino acid changes in the antigenic sites. Oligosaccharides which are recognised as "self" by the host immune system may pose conformational changes in the molecule and mask antigenic sites, thereby prevent binding of host antibodies. The number of N-linked glycosylation sites in the H3 HA protein has increased during the years from only three attachment sites in 1968 [58,59] to ten predicted sites in the Danish isolates after 2004.

The A/Fujian/411/02(H3N2)-like stains from the 2002–2003 season gained a potential glycosylation site at position 144, thereby masking the supposed "key" site for antigenic change [33]. Substitutions at antigenic site B and the predicted N-glycosylation at position 144 in HA antigenic site A together with a stronger NA might have contributed to the increased infectivity of the reassorted Fujian-like viruses of the 2003–2004 season, causing an epidemic in Denmark. We have shown that the preferred antibody epitope for genetic change is antigenic site B for the Danish dataset also reflecting that site A is camouflaged by glycosylation. Thus the antigenic changes at a glycosylated site A may not play a major role in escape from the immune system as long as the glycosylation is present.

Six potential N-glycosylation sites have been conserved in the N2 NA Danish dataset from 1999 to 2003. The majority of isolates from 2003 to 2006 have lost the site at position 93 which is located in a CTL epitope (HLA-A*0201) region of NA [12]. The recent NAs may therefore be more easily recognised by the cytotoxic immune system. We found two predicted N-glycosylation sites (61 and 70) in the N2 NA stalk region in sequences from 1999–2006. Greater density of carbohydrate in the stalk region of NA might reflect a need for proteolytic protection. The two observed carbohydrates in the stalk have been reported for other isolates in other time periods and other continents [58,60]. The stalk region has therefore stayed unchanged and the two sequons seem to be conserved.

### Sequence data

As expected a higher dN/dS ratio was observed for the surface glycoproteins, although none were directly influenced by positive selection. We observed that the HA1 region and the M2 protein have a slightly higher global dN/dS ratio than the other genes (Table 3). This is consistent with the findings of others [11,61]. The M2 protein is a membrane ion channel protein on the surface of the virus molecule, a higher dN/dS ratio for this protein compared to the internal proteins is expected. The ratio might, however, be biased because the M2 protein is spliced from M and the dS is suppressed for overlapping regions giving a higher dN/dS ratio [11].

Earlier findings support positive selection at sites involved in receptor and antigen binding [11,62]. Five of the 18 codons in HA proposed by Bush *et al*., [63] to be under positive selection were found to have changed in our HA dataset of H3N2 since 1999, namely: pos 145, 156, 186, 193 and 226. In our H3 dataset (n = 204) site 199 was influenced by positive selection as calculated by FEL analysis, but no positively selected sites were found applying the more conservative SLAC method. Position 199 may be involved in receptor binding and influence on the virus ability to grow in eggs [10]. In a similar study on a slightly larger dataset (n = 284) positions 220 and 229 were found to be positively selected [11]. Another study found positions 13 and 236 [62]; however, suggested positively selected sites may vary by the dataset applied, method used and the significance level selected for a site to be classified as positively selected. REL analysis identified four sites (208, 211, 218 and 219) in M1 under positive selection pressure. REL analysis tends to give better estimates on small datasets than SLAC and FEL. Sites 211, 218, and 219 were still selected when the bayes factor cut-off was increased from 50 to 200. Further analysis would be needed to determine if these sites actually are positively selected.

## Conclusion

There is a need for complete genome analysis of European human influenza A viruses in order to gather a comprehensive picture of the evolution and migration of viruses. Our results support the suggestion that the evolution of influenza A viruses is more complex than originally believed [28,62]. Local short term evolution of influenza virus is a stochastic process, also involving gene reassortments. It will be interesting to further investigate how viruses from other parts of Europe influence on the evolution of Danish isolates when more full length sequences from Europe are made public.

## Methods

### Human samples

A total of 234 Danish human nasal swab suspensions or nasopharyngeal aspirates positive for influenza A, from 1999 to 2006, were available at the WHO National Influenza Centre, Copenhagen. The seasonal distribution was as follows: 1999–2000 15 samples, 2000–2001 13 samples, 2001–2002 10 samples, 2002–2003 30 samples, 2003–2004 76 samples, 2004–2005 51 samples and 2005–2006 39 samples. Based on the phylogenetic clustering of all HAs and NAs, ten representative samples from each season and each subtype, if possible, were selected for further analysis.

### RNA extraction and full-length one-step RT-PCR

Viral RNA was extracted from 140 μl of human nasal swab suspension or nasopharyngeal aspirate by QIAamp^® ^Viral RNA Mini Kit (QIAGEN, Germany) as described by the manufacturer or by an automated MagNA Pure LC Instrument applying the MagNa Pure LC Total Nucleic Acid Isolation Kit (Roche Diagnostics, Basel, Switzerland). The different gene segments were amplified by OneStep RT-PCR Kit (QIAGEN) as previously described [64], applying a two minute elongation step for all genes. The primers for RT-PCR were segment specific but subtype universal targeting the highly conserved noncoding RNA regions at the 5'- and 3'-end of each segment [65]. PCR products were purified using the GFX™ PCR DNA and Gel Band Purification Kit (Amersham Biosciences, Germany) prior to sequencing.

### Sequencing and phylogenetic analyses

Purified PCR products were sequenced directly. Primer sequences are available upon request. The sequencing reaction was performed by ABI PRISM^® ^BigDye™ Terminators v3.1 Cycle Sequencing Kit (Applied Biosystems, USA) as described previously [66]. The sequences were developed on an automatic ABI PRISM^® ^3130 genetic analyzer (Applied Biosystems) with 80 cm capillaries. Consensus sequences were generated in SeqScape^® ^Software v2.5 (Applied Biosystems). Sequence assembly, multiple alignment and alignment trimming were performed with the BioEdit software v.7.0.5 [67]. Distance based neighbor joining (NJ) phylogenetic trees and character based maximum parsimony (MP) trees were generated using the Molecular Evolutionary Genetics Analysis (MEGA) software v.3.1 [68]. Maximum likelihood trees were generated by the Phylogenetic Analysis Using Parsimony (PAUP 4.0) Software (Sinauer Associates, Inc.) [69] applying the HKY85 nucleotide model, allowing transitions and transversions to occur at different rates.

### Sequence data

The between-seasons nucleotide distance means were computed as the arithmetic mean of all pair wise distances between two seasons in the inter-season comparisons using the MEGA v.3.1 software [68]. The global rate between dN and dS substitutions and the individual site-specific selection pressure were measured by the likelihood based single likelihood ancestor counting (SLAC) method in Datamonkey (modified Suzuki-Gojobori method) [70,71]. For datasets over 100 sequences (H3 n = 204 and N2 n = 166) the HyPhy package [72] was applied. The estimations are likelihood-based, employing a codon model cross between HKY85 and MG94. To elucidate single, positively selected amino acids, the HA and NA datasets were analysed with SLAC and a two rate fixed effects likelihood (FEL) [73] using a likelihood approach with neighbor joining phylogenetic trees (HyPhy). Sites with dN>dS with a <0.05 significance level for likelihood ratio test (LRT) were implied as positively selected for the large HA and NA datasets. For the small datasets (genes coding for the internal proteins <15 and the H1N1 viruses <30) we additionally ran a random effects likelihood (REL) analysis using an empirical Bayes approach with NJ phylogenetic trees in Datamonkey [70]. This method is expected to calculate positively selected sites more accurately in small datasets. The accepted significance level for a positively selected site was set at <0.1 (two-tailed binominal distribution) for SLAC and FEL analyses and >50 bayes factor for REL.

### Prediction of glycosylation sites

Potential N-linked glycosylation sites were predicted using nine artificial neural networks with the NetNGlyc 1.0 Server [74]. A threshold value of >0.5 average potential score was set to predict glycosylated sites. The N-Glycosite prediction tool at Los Alamos [75] was used to visualise the fraction of isolates possessing certain glycosylated sites along the sequence alignment.

### Calculation of antigenic distance

The specific measure of antigenic distance between two strains of influenza were calculated as *P*_epitope _values by the method suggested by Muñoz, *et al.*, [39]. The *P*_epitope _value was calculated as the number of mutations within an antibody antigenic site divided by the number of amino acids defining the site. It is assumed that an antigenic epitope which has the greatest percentage of mutations is dominant, because that epitope is influenced by the greatest selective pressure from the immune system. The *P*_epitope _distance is defined as the fractional change between the dominant antigenic epitopes of one strain compared to another. The *P*_epitope _Calculator [76,77] was applied for H3 sequences. Residues in antigenic epitopes were collected from several references [9,13,39,40,48,78,84].

### Nucleotide sequence accession numbers

Nucleotide sequence accession numbers submitted to GenBank are; H3: AY531039–AY531046, AY531048–AY531049, AY531051–AY531052, AY531054, AY531056–AY531057, EU103820–EU103823, EU103631–EU103819, N2: AY531006–AY531013, AY531015–AY531016, AY531018–AY531020, AY531023, AY531026–AY531028, EU103825–EU103977, H1: EU097933–EU097962, EU103824, N1: EU097708–EU097737, PB2: EU097738–EU097768, PB1: EU097769–EU097801, PA: EU097802–EU097833, NP: EU097834–EU097866, M: EU097867–EU097899, NS: EU097900–EU097932].

## Competing interests

The author(s) declare that they have no competing interests.

## Authors' contributions

KB conceived and designed the experiments. KB performed the experiments, data analysis and wrote the paper. LPN and AF contributed reagents and materials. AFO supervised the research. Further LPN and AF critically revised the manuscript and AF gave the final approval for publication. All authors read and approved the final manuscript.
